# Epigenetic Markers of Renal Function in African Americans

**DOI:** 10.1155/2013/687519

**Published:** 2013-12-12

**Authors:** Samantha M. Bomotti, Jennifer A. Smith, Alicia L. Zagel, Jacquelyn Y. Taylor, Stephen T. Turner, Sharon L. R. Kardia

**Affiliations:** ^1^Department of Epidemiology, Bloomberg School of Public Health, Johns Hopkins University, Baltimore, MD 21205, USA; ^2^Department of Epidemiology, School of Public Health, University of Michigan, 1415 Washington Heights, No. 4629, Ann Arbor, MI 48109, USA; ^3^Center for Health Statistics, Washington State Department of Health, Olympia, WA 98501, USA; ^4^School of Nursing, Yale University, New Haven, CT 06477, USA; ^5^Division of Nephrology and Hypertension, Mayo Clinic, Rochester, MN 55905, USA

## Abstract

Chronic kidney disease (CKD) is an increasing concern in the United States due to its rapidly rising prevalence, particularly among African Americans. Epigenetic DNA methylation markers are becoming important biomarkers of chronic diseases such as CKD. To better understand how these methylation markers play a role in kidney function, we measured 26,428 DNA methylation sites in 972 African Americans from the Genetic Epidemiology Network of Arteriopathy (GENOA) study. We then evaluated (1) whether epigenetic markers are associated with estimated glomerular filtration rate (eGFR), (2) whether the significantly associated markers are also associated with traditional risk factors and/or novel biomarkers for eGFR, and (3) how much additional variation in eGFR is explained by epigenetic markers beyond established risk factors and biomarkers. The majority of methylation markers most significantly associated with eGFR (24 out of the top 30) appeared to function, at least in part, through pathways related to aging, inflammation, or cholesterol. However, six epigenetic markers were still able to significantly predict eGFR after adjustment for other risk factors. This work shows that epigenetic markers may offer valuable new insight into the complex pathophysiology of CKD in African Americans.

## 1. Introduction

Chronic kidney disease (CKD) is an increasing public health concern in the United States due to its rapidly rising incidence and prevalence, particularly among older individuals. While about 20 million United States adults over the age of 20 (10%) currently have CKD, the prevalence of CKD among those 60 and older is approximately 25% [1]. Further, the incidence of CKD among those aged 65 and older more than doubled between 2000 and 2008 [1]. As a result, health care costs related to the most severe form of CKD, End-Stage Renal Disease (ESRD), have also nearly doubled in the past decade to over $40 billion per year [1]. Individuals over 60 are almost 6 times more likely to develop CKD than those aged 20–39, and females are 1.4 times more likely than males to develop it [2]. Further, African Americans are at higher risk for ESRD than other races. While African Americans accounted for only 12% of the US population in 2009, they accounted for nearly one-third of kidney failure cases [3].

Level of kidney function is assessed by the glomerular filtration rate (GFR), the rate at which blood passes through the kidney's filtering mechanisms. GFR levels below 60 mL/min/1.732 m^2^ are used, in conjunction with markers of kidney damage such as proteinuria, to diagnose CKD and determine its severity [4]. GFR is difficult to measure directly, but it can be estimated using blood markers such as creatinine in combination with demographic factors (age, sex, and ethnicity). Since the early stages of CKD generally have few or no symptoms, disease detection is difficult before progressive kidney damage has already occurred [5].

Several risk factors have been implicated in CKD etiology. The most common are diabetes mellitus, hypertension, obesity, elevated cholesterol, smoking, and cardiovascular disease (CVD) [3, 5]. Approximately 20% and 35% of US adults with diabetes and hypertension, respectively, have CKD [3]. Further, a recent study showed that hypertension, smoking, obesity, and low HDL cholesterol were associated with reduced kidney function [6]. While risk prediction of CKD is in its infancy, studies have found moderate prediction capability for CKD development and progression using models that include hypertension, diabetes, history of CVD, body mass index, and other variables [7].

The identification of new biomarkers for early detection of CKD is crucial to developing novel prevention strategies. It is particularly important to identify markers within high-risk populations in order to reduce social disparities in CKD and ESRD incidence. Researchers have been exploring the use of inflammatory markers as potential biomarkers of kidney function in recent years. Several such markers have been shown to be strongly associated with renal function. Specifically, C-reactive protein, fibrinogen, homocysteine, and several other markers of inflammation are elevated in individuals with decreased kidney function [8–11].

Epigenetic markers are now also being considered as potentially viable predictors of kidney function [12]. Epigenetics refers to mitotically heritable genomic modifications that do not alter the underlying DNA sequence. DNA methylation is one type of epigenetic modification that alters gene transcription [13], potentially influencing initiation and progression of chronic diseases such as CKD. Alterations in DNA methylation have already been shown to be associated with a variety of chronic diseases including CKD, cardiovascular disease, cancer, diabetes, and systemic lupus erythematosus [10, 12–21]. For example, a recent study found that patients with Stage 5 CKD with inflammation and hyperhomocysteinemia exhibited global DNA hypermethylation in blood leukocytes compared to patients with no inflammation and typical homocysteine levels [10]. In spite of the progress in this research area, little is still known about the relationship between epigenetics and kidney disease.

In order to better understand how epigenetics, inflammation, and traditional risk factors can explain the variation in kidney function, we conducted a study that evaluated three key questions: (1) is DNA methylation in peripheral blood cells associated with eGFR? (2) are the significantly associated epigenetic markers also associated with traditional risk factors and/or novel biomarkers for eGFR? and (3) how much additional variation in eGFR is explained by the epigenetic markers beyond these risk factors and biomarkers? Although peripheral blood cells may not be fully representative of kidney epigenetic patterns, leukocytes (a key component of peripheral blood cells) orchestrate the inflammatory responses within the kidney and are therefore likely to play a role in the pathophysiology of CKD. Juxtaposing the epigenetic biomarkers in peripheral blood cells against more traditional risk factors will allow us to identify new interconnections between genome biology and CKD precursors.

## 2. Methods

### 2.1. Sample

The Genetic Epidemiology Network of Arteriopathy (GENOA) study is a community-based study investigating the genetics of hypertension and its arteriosclerotic complications in non-Hispanic whites from Rochester, Minnesota, and African Americans from Jackson, Mississippi [22]. In this study, we investigated the relationship between DNA methylation and eGFR in GENOA African Americans. African American sibships in which at least two siblings had been diagnosed with primary hypertension before the age of 60 (*N* = 1, 854) were recruited for an initial examination (Phase I: 1996–1999) that included standardized interviews concerning demographic and medical history, as well as a physical examination and blood sample collection. The second examination (Phase II: 2000–2004) comprised 1,482 participants returning from Phase I. This exam included re-assessment of the original interview, a physical examination, and a blood draw, as well as additional measurements of arteriosclerotic target-organ damage of the kidney, heart, brain, and peripheral arteries.

### 2.2. Measurement of Traditional Risk Factors

Height was measured by stadiometer and weight by electronic balance, and body mass index (BMI) was calculated as weight in kilograms divided by the square of height in meters. Resting systolic blood pressure and diastolic blood pressure were measured by a random zero sphygmomanometer. The diagnosis of hypertension was established based on BP levels measured at the study visit (>140 mmHg average systolic BP or >90 mmHg average diastolic BP) or a prior diagnosis of hypertension and current treatment with antihypertensive medications.

Blood was drawn by venipuncture after an overnight fast. Serum triglycerides (TG), creatinine, total cholesterol, glucose, and high-density lipoprotein (HDL) cholesterol were measured by standard enzymatic methods on a Hitachi 911 Chemistry Analyzer (Roche Diagnostics, Indianapolis, IN). Estimated GFR (eGFR) was calculated for each participant from serum creatinine values and relevant demographic factors (age, sex, and race) using the CKD-EPI creatinine equation [23]. Diagnosis of diabetes was established based on fasting glucose levels >126 mg/dL measured at the study visit or current treatment with diabetes medications. C-reactive protein was measured by a highly sensitive immunoturbidimetric assay [24], fibrinogen was measured by the Clauss (clotting time based) method [25], and plasma homocysteine was measured by high-pressure liquid chromatography.

### 2.3. Measurement of DNA Methylation

DNA methylation was quantified on 1,008 Phase II participants using stored blood samples collected during the second examination. Samples were prepared and DNA methylation was measured according to previously published methods [26–28]. Briefly, the Illumina Infinium HumanMethylation27 BeadChip microarray was used to measure DNA methylation at 27,578 CpG sites. To reduce batch and chip effects, the correlation structure among 56 control probes was evaluated within channel to identify the most parsimonious subset of probes that explained the maximum amount of batch and chip variation across samples (5 probes in the red channel and 8 probes in the green channel). Normalization was conducted by linearly regressing the 13 selected probes onto the intensity signals from the methylated (**M**) and unmethylated (**U**) bead types separately across each CpG site.

The *M*-value is a commonly used measurement in microarray analysis that was recently adapted for use with DNA methylation array data. We chose to assess DNA methylation using the *M*-value because the statistical distributions of *M*-values for individual CpG sites conform to modeling assumptions more often than do those of other standard metrics, such as the Beta value [29, 30]. The *M*-value for each individual *i* at a single site, *k*, is calculated as follows: *M*-value_*ik*_ = log⁡_2_⁡ [(max⁡(**M**_*ik*_, 0) + 1)/(max⁡(**U**_*ik*_, 0) + 1)] [30]. *M*-values that are <−2.0 are considered to be unmethylated, *M*-values that are >2.0 are considered methylated, and *M*-values that are between −2.0 and 2.0 are considered semimethylated.

Prior to statistical analysis, we removed samples that had poor bisulfite conversion (*N* = 7), as determined by bisulfite-conversion control fluorescence intensity of <4,000. An additional 29 samples were removed from the analysis due to extreme control probe values, assessed as having at least one control probe with a value of greater than 4 standard deviations from its mean value. This resulted in a total sample size of 972 individuals. 

In this study, we analyzed only autosomal CpG sites. A total of 58 CpG sites were removed from the analysis because they were found to be multimodal based on the Dip Test proposed by J. A. Hartigan and P. M. Hartigan [31] using a cut-off of *P* < 0.001 on the signal intensities of the methylated and/or unmethylated bead types. This resulted in a total number of 26,428 CpG sites included in our analysis.

### 2.4. Statistical Analyses

#### 2.4.1. Linear Mixed Modeling

We used linear mixed modeling to identify the top 30 CpG sites that were most significantly associated with eGFR, prior to adjustment for any risk factors. Rather than adjusting for age and sex prior to any statistical analysis, each of the 26,428 CpG sites were modeled individually as covariates against eGFR so that we were not failing to detect sites that act as mediators of age and sex on eGFR *a priori*. The linear mixed model eGFR_*ij*_ = *β*_0_ + *β*_1_ · CpG_*ij*_ + *W*_*j*_ + *ε*_*ij*_ (Model 1) was estimated using the nlme package in the statistical R software version 2.14.0 [32]. CpG_*ij*_ is the *M*-value of the epigenetic marker for participant *i* in sibship *j*, and *W*_*j*_ is the random effect for sibship *j*.

#### 2.4.2. Linear Modeling

In order to determine the risk factors most highly associated with eGFR, we performed forward selection using traditional linear modeling. We then checked the robustness of the modeling using linear mixed modeling to ensure that accounting for family structure did not influence the inferences of the linear modeling. We used traditional linear modeling instead of linear mixed modeling to facilitate the comparison of *R* ^2^ values among nested models. Univariable linear regression models of the generic form eGFR_*i*_ = *β*_0_ + *β*_1_ · Risk  Factor_*i*_ + *ε*_*i*_ (Model 2) were used to evaluate the relationships between eGFR and traditional risk factors as well as novel risk factors. Traditional risk factors including diabetes, hypertension, cholesterol, blood pressure, age, sex, and anthropometric measures were considered in addition to novel inflammatory biomarkers such as homocysteine, fibrinogen, and C-reactive protein. All significantly associated risk factors were then processed in forward selection methods with an entry significance level of *P* < 0.05 in SASv9.3 (SAS Institute Inc., Cary, NC) to determine the amount of variation in eGFR explained by all of the risk factors in the model.

Univariable linear models of the generic form Risk  Factor_*i*_ = *β*_0_ + *β*_1_ · CpG_*i*_ + *ε*_*i*_ (Model 3) were used to determine whether there were any associations between the risk factors identified in Model 2 and each of the top 30 significant CpG sites for eGFR identified in Model 1. Once all of the CpG sites significantly associated with each risk factor were determined, bivariable linear models of the form eGFR_*i*_ = *β*_0_ + *β*_1_ · Risk  Factor_*i*_ + *β*_2_ · CpG_*i*_ + *ε*_*i*_ (Model 4) were used to estimate the contribution of each CpG site that remained significantly associated with eGFR after the adjustment for each risk factor.

The multivariable model eGFR_*i*_ = *β*_0_ + ∑_*m*=1_^*p*^ *β*_*m*_ · Risk  Factor_*mi*_ + *ε*_*i*_ (Model 5) estimated by forward selection procedures (previously described) allowed us to estimate how much of the eGFR variation could be explained by risk factors. The multivariable model eGFR_*i*_ = *β*_0_ + ∑_*m*=1_^*p*^ *β*_*m*_ · Risk  Factor_*mi*_ + *β*_*p*+1_ · CpG_*i*_ + *ε*_*i*_ (Model 6) was then used to investigate whether each individual epigenetic marker added additional predictive information about eGFR. The final model combining risk factors and epigenetic markers was eGFR_*i*_ = *β*_0_ + ∑_*m*=1_^*p*^ *β*_*m*_ · Risk  Factor_*mi*_ + ∑_*q*=*p*+1_^*p*+*r*^ *β*_*q*_ · CpG_*qi*_ + *ε*_*i*_ (Model 7). This model was estimated using forward regression techniques (keeping the risk factors constant from previous models).

## 3. Results

### 3.1. Descriptive Statistics

A majority of the study population was female (71%), with a mean age of 66.3 years. Many of the participants had hypertension (83%) and/or diabetes (31%). Less than half of the participants had ever smoked (42%) and the mean BMI was 31.2 kg/m^2^ (Table 1). Women had a higher average BMI and higher levels of cholesterol, HDL cholesterol, C-reactive protein, and fibrinogen than men, and they had lower diastolic blood pressure and homocysteine levels. A majority of the 26,428 CpG sites evaluated in this study had mean values that were considered unmethylated: 58% of CpG sites (15,217 sites) had average *M*-Values <−2.0 (see Figure 1 in Supplementary material available online at http://dx.doi.org/10.1155/2013/687519).

### 3.2. Top 30 CpG Sites Associated with eGFR

Nineteen methylation markers were significantly associated with eGFR at the Bonferroni-corrected *P* value for an alpha level of 0.05 (1.89 × 10^−6^) (see Supplementary Figure 2 for a Q-Q plot of the results from the association between genome-wide CpG sites and eGFR). However, the highly intercorrelated nature of the epigenomic markers renders Bonferroni correction too conservative. Most of the top 30 CpG sites were positively associated with eGFR (Table 2) and the majority of these sites have at least moderate correlation >0.40 with at least one of the other 29 CpG sites (Supplementary Table 1). Thus, we chose to evaluate the top 30 most strongly associated sites, all of which had *P* values less than 6 × 10^−6^. The top 30 CpG sites explain 13% (*R* ^2^ = 0.13) of the variation in eGFR collectively based on simple linear regression.

### 3.3. Risk Factors for eGFR

**Figure 1:**
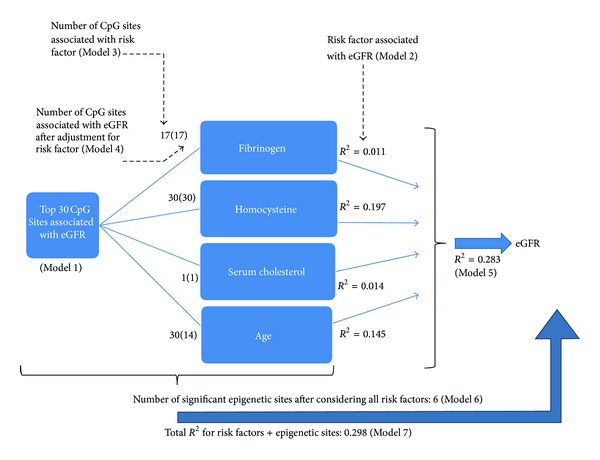
Flow chart of the contributions of all models used in this study and their relationships with eGFR.

In order to compare the effects of epigenetic markers relative to traditional biomarkers/risk factors, we first performed univariable and then multivariable modeling of eGFR (Table 3). Fibrinogen, homocysteine, serum cholesterol, and age were the four risk factors that were found using forward selection to be significantly associated with eGFR at the significance threshold *α* = 0.05 (Table 3). Homocysteine and age explained the largest amount of variability in eGFR (*R* ^2^ = 0.197 and 0.145, resp.) (Figure 1). All four risk factors together explained a total of 28.3% of the variation in eGFR.

### 3.4. Association of Risk Factors of eGFR with Top 30 Methylation Markers

To better understand whether the epigenetic sites are operating independently or through risk factors, the top 30 CpGs were then incorporated individually into models with the four significant risk factors as outcomes (Model 3) to examine the associations between each methylation marker and the four risk factors individually (Supplementary Table 2). Seventeen of the thirty CpG sites were significantly associated with fibrinogen, one was significantly associated with serum cholesterol, and all thirty were significantly associated with homocysteine and age, all at *α* = 0.05. Since all of the top sites were found to be associated with at least one of the four risk factors for eGFR when placed in univariate models against each risk factor (Figure 1, Supplementary Table 3), the next step was to investigate whether the epigenetic sites remained significant predictors of eGFR after adjustment for the aforementioned risk factors.

### 3.5. Explanation of Variation in eGFR by Methylation Markers after Adjustment

Fourteen of the thirty CpG sites associated with age remained significantly associated (*P* < 0.05) with eGFR in the bivariable model (Figure 1, Supplementary Table 3). All of the CpG sites that were associated with the other significant risk factors (fibrinogen, serum cholesterol, and homocysteine) remained significantly associated with eGFR in the bivariable model, indicating that these epigenetic markers are independent predictors of kidney function. Individual CpG sites were able to predict an additional 1–4% of the variation in eGFR beyond individual risk factors.

### 3.6. Explanation of Variation in eGFR by Methylation Markers after Adjustment for Risk Factors

In order to determine whether the CpG sites remained significant predictors of eGFR after adjusting for all four risk factors, we constructed multivariable models. A multivariable model with the four risk factors and a single CpG site predicting eGFR (Model 6) was compared to the multivariable model with only the four risk factors (Model 5). This was repeated for each of the 30 CpG sites. Six CpG sites remained significantly associated with eGFR after adjustment for all four risk factors and predicted approximately 0.3–0.8% of the variation in eGFR beyond the risk factors (Figure 1, Supplementary Table 4).

We used forward selection to build a model that consisted of the four risk factors plus the CpG sites that remained significant predictors of eGFR (Model 7). Three of the six CpG sites (cg26842024, cg07426848, and cg17589341) remained significant in the final model in addition to the established risk factors (Supplementary Table 5). The final model was able to predict 29.8% of the variation in eGFR (Figure 1).

## 4. Discussion

The purpose of this study was to identify methylation sites in peripheral blood cells that were significantly associated with eGFR, to evaluate their association with CKD risk factors, and to determine whether these epigenetic sites were still predictors of eGFR after adjustment. By evaluating DNA methylation within peripheral blood cells, we were able to examine the relationship between kidney function and epigenetic processes occurring within cells that are involved in inflammatory responses in the kidney. Given the large number of correlated epigenetic sites, we focused our study on the top 30 CpG sites that were significant after adjusting for multiple testing.

We used this approach to identify the epigenetic markers of eGFR because previous studies have indicated that many epigenetic sites are associated with age and that these sites could potentially provide fundamental insights into the biology of the aging kidney. Indeed, the top 30 CpG sites were all significantly associated with age in our study. However, 14 of these 30 CpG sites remained significant predictors of eGFR after adjustment for age. These same 30 CpG sites were also significantly associated with plasma levels of homocysteine in our study, and all 30 CpG sites remained significant predictors of eGFR after adjustment for homocysteine (a significant predictor of eGFR). Only six of the 30 CpG sites remained significant predictors of eGFR after adjustment for traditional risk factors (age, fibrinogen, serum cholesterol, and homocysteine). Consequently, it appears that the majority of the epigenetic markers that we identified may affect eGFR, at least in part, through pathways related to aging, inflammation, and cholesterol.

As a final step, we put all the top epigenetic sites and risk factors into a forward selection algorithm and identified three significant, independent CpG site predictors of eGFR. The three significant CpG sites are cg26842024 in *KLF2* gene, cg07426848 in the *S100A3* gene, and cg17589341 in the *SLC14A1* gene. The *KLF2* gene encodes a Krüppel-like transcription factor. This zinc finger family of transcription factors is important in regulating cellular processes in the vasculature, including the kidney's glomerular capillary bed [33]. For example, in renal transplants with thrombotic microangiopathy, studies of gene expression in the glomerulus have demonstrated a downregulation of *KLF2* and subsequent upregulation of genes that inhibit local fibrinolysis [34]. Recently, researchers have shown that the impact of blood flow and its laminar shear stress on glomerular endothelial cells alters expression of *KLF2*. Specifically, chronic laminar shear stress increases *KLF2* which then increases expression of endothelial nitric oxide synthase (eNOS), thrombomodulin, and nitric oxide [35]. This study also demonstrated that these changes in the glomerular endothelium associated with *KLF2* had an effect on kidney podocytes. Podocytes are cells in the glomerulus responsible for the kidney's ability to filter waste products from the blood and are intimately involved in the pathogenesis of CKD [36].

The other two significant CpG sites are within genes that are related to bladder biology and, hence, may reflect downstream consequences of variability in eGFR in our study. That is, differences in methylation within these genes may be a response to higher or lower levels of eGFR rather than influencing eGFR itself. In particular, the *S100* proteins are signaling factors that are involved in regulation of cellular processes in a wide range of cell types. The differential expression of *S100A3* has been implicated in the bladder cancers [37]. The solute carrier family 14 (urea transporter), member 1 (*SLC14A1*) gene has been studied for decades as the Kidd blood group. This urea transporter is expressed in a wide range of cell types. Recently, genetic studies have identified it as an important gene involved in the concentration of the urine in the kidney [38] as well as an important susceptibility gene for bladder cancer [39, 40].

These three genes that were significant independent predictors of eGFR point to future studies that may help to understand the mechanism underlying interindividual variation in kidney function in African Americans. We are unaware of other studies that have investigated the epigenetic predictors of eGFR on a genome-wide scale. However, a few other studies of kidney-related diseases point to the breadth of epigenetic studies of kidney phenotypes that could be conducted. For example, a study on the epigenetic markers of diabetic nephropathy among African Americans and Hispanics identified 187 genes that were differentially methylated among diabetes patients with and without nephropathy [16]. Another study found that epigenetic “metabolic memory” from prior exposure to hyperglycemia, even after glucose normalization, was implicated in End-Stage Renal Disease among African American diabetic patients [12].

Our study has several notable strengths including a large sample size, investigation of a key indicator of kidney function in a minority population, and epigenome-wide assessment of DNA methylation. However, it also has several limitations. First, the GENOA sample does have an increased prevalence of hypertension compared to an unselected population of the same age range. Hypertension is associated with measures of eGFR, and thus the distribution of eGFR in this sample differs from that of an unselected population. Also, the current study was cross-sectional rather than longitudinal, so we cannot discern the temporal relationship between changes in DNA methylation and changes in inflammatory processes that influence kidney function.

This study and other epigenetic studies support the idea that differential DNA methylation in peripheral blood cells may be an indicator of kidney function and may potentially help us understand etiological aspects of kidney disease because it provides an important link to inflammatory processes that underlie chronic diseases such as CKD. Better understanding of the role of epigenetics in kidney function, particularly among African Americans, may lead to the development of novel detection, treatment, or prevention strategies for CKD that will help to decrease the current health disparities in kidney disease.

## Supplementary Material

The supplementary material includes five tables and two figures that further characterize the relationship between DNA methylation markers, traditional risk factors for kidney function, and estimated glomerular filtration rate (eGFR). Specifically, the tables show the correlation between the 30 methylation sites most strongly associated with eGFR (Supplementary Table 1), the relationship between these 30 methylation sites and the four risk factors that are most strongly associated with eGFR (Supplementary Table 2), the variation in eGFR that is explained by each of the 30 methylation sites after adjusting for each risk factor individually (Supplementary Table 3), the variation in eGFR that is explained by each of the 30 methylation sites after adjusting for all significant risk factors (Supplementary Table 4), and the variation in eGFR that is explained by the final model that includes the significant risk factors and methylation sites (Supplementary Table 5). Supplementary Figure 1 shows the distribution of the M-value for the 26,428 DNA methylation sites that were tested for association with eGFR. Supplementary Figure 2 shows the quantile-quantile plot of the association between each of the 26,428 DNA methylation sites and eGFR, without adjustment for any risk factors.

## Figures and Tables

**Table 1:** Descriptive statistics of traits studied in GENOA African Americans.

	Total *N* = 972	Males *N* = 285	Females *N* = 687	*P* value
	Mean (SD)	Mean (SD)	Mean (SD)	
Estimated glomerular filtration rate (mL/min per 1.732 m^2^)	85 (21)	83 (21)	86 (21)	0.0646^a^
Age (years)	66 (8)	67 (8)	66 (8)	0.2179^a^
Height (cm)	167 (9)	178 (6)	164 (6)	3.1*E* − 153^a^
Weight (kg)	88 (18)	92 (17)	86 (18)	3.3*E* − 05^a^
Waist circumference (cm)	104 (14)	104 (13)	103 (15)	0.7565^a^
Hip circumference (cm)	116 (14)	110 (11)	118 (15)	1.3*E* − 17^a^
Body mass index (kg/m^2^)	31 (6)	29 (5)	32 (7)	1.7*E* − 15^a^
C-reactive protein (mg/L)	0.7 (1)	0.6 (1)	0.7 (1)	0.00003^b^
Fibrinogen (mg/dL)	369 (82)	354 (87)	376 (80)	0.0002^a^
Homocysteine (*μ*mol/L)	11 (5)	12 (5)	10 (4)	4.7*E* − 09^b^
Serum cholesterol (mg/dL)	204 (42)	192 (40)	209 (42)	1.8*E* − 08^a^
Serum glucose (mg/dL)	113 (44)	116 (51)	112 (40)	0.3147^b^
Serum triglycerides (mg/dL)	120 (64)	120 (74)	120 (60)	0.3292^b^
Systolic blood pressure (mm Hg)	140 (21)	138 (21)	141 (22)	0.0396^a^
Diastolic blood pressure (mm Hg)	78 (11)	80 (11)	78 (11)	0.0005^a^
Combined high-density lipoprotein (mg/dL)	58 (18)	50 (15)	62 (18)	5.6*E* − 24^b^

	*N* (%)	*N* (%)	*N* (%)	*P* value^c^

Hypertension	802 (83)	228 (80)	574 (84)	0.1845
Diabetes	298 (31)	86 (30)	212 (31)	0.8334

^a^ *P* value from an independent *t*-test of means between males and females for normally distributed continuous variables.

^b^ *P* value from a two-sided *Z*-test of a nonparametric Wilcoxon test for nonnormally distributed continuous variables.

^c^ *P* value from a chi-square test for categorical variables.

**Table 2:** Top 30 CpG sites most strongly associated with eGFR (Model 1).

	Genetic description			*M*-value information		
CpG Site	Chr	Gene	Gene product	*M*-value mean (SD)	*β*_1_	*P*-value
cg00226923	6	*FGD2 *	FYVE; RhoGEF and PH domain containing 2	2.9 (0.4)	−8.8	6.1*E* − 09
cg17471102	19	*FUT3 *	Galactoside 3(4)-L-fucosyltransferase	0.7 (0.3)	12.4	1.3*E* − 08
cg12261786	10	*ADIRF *	Adipogenesis regulatory factor	1.2 (0.3)	12.6	2.7*E* − 08
cg10917602	16	*HSD3B7 *	3 Beta-hydroxy-delta 5-C27-steroid oxidoreductase	0.4 (0.4)	8.3	6.7*E* − 08
cg04662594	8	*EPB49 *	Erythrocyte membrane protein band 4.9 (dematin)	−0.8 (0.4)	9.6	8.8*E* − 08
cg15121304	22	*IGL2 *	Immunoglobulin lambda locus	1.9 (0.3)	−10.7	1.8*E* − 07
cg24857721	1	*RHD *	Rh blood group D antigen isoform 1	0.4 (0.4)	8.5	2.4*E* − 07
cg14688272	17	*FN3KRP *	Fructosamine-3-kinase-related protein	−0.2 (0.3)	11.3	2.9*E* − 07
cg19761273	17	*HCKID *	Casein kinase 1; delta isoform 1	−2.0 (0.3)	10.9	4.6*E* − 07
cg24092253	20	*YTHDF1 *	YTH domain family; member 1	−1.2 (0.3)	11.0	5.8*E* − 07
cg10126923	19	*NKG7 *	Natural killer cell group 7 sequence	−0.2 (0.5)	6.6	6.1*E* − 07
cg25538571	8	*FLJ46365 *	Hypothetical protein LOC401459	−0.7 (0.3)	10.8	6.3*E* − 07
cg11120551	1	*CHD1L *	Chromodomain helicase DNA binding protein 1-like	−0.9 (0.4)	8.9	7.6*E* − 07
cg00563932	9	*PTGDS *	Prostaglandin H2 D-isomerase	0.3 (0.3)	9.7	8.9*E* − 07
cg16280667	11	*BLR1 *	Burkitt lymphoma receptor 1 isoform 1	2.1 (0.4)	−9.4	1.1*E* − 06
cg12125117	16	*GPR97 *	G protein-coupled receptor 97	−0.8 (0.5)	7.0	1.4*E* − 06
cg01820374	12	*LAG3 *	Lymphocyte-activation protein 3 precursor	−0.7 (0.3)	10.2	1.5*E* − 06
cg09809672	1	*EDARADD *	EDAR-associated death domain isoform B	−0.4 (0.4)	7.5	1.7*E* − 06
cg14859417	10	*PTPRE *	Protein tyrosine phosphatase; receptor type; E isoform 2	−1.7 (0.4)	9.1	1.9*E* − 06
cg18152830	17	*TNFRSF13B *	Tumor necrosis factor receptor 13B	2.6 (0.3)	−10.8	2.2*E* − 06
cg08743392	20	*GSS *	Glutathione synthetase	−2.5 (0.4)	8.3	2.3*E* − 06
cg26842024	19	*KLF2 *	Kruppel-like factor 2	−2.9 (0.4)	−8.2	2.4*E* − 06
cg07426848	1	*S100A3 *	S100 calcium binding protein A3	2.1 (0.3)	−9.5	2.6*E* − 06
cg15297650	2	*DKFZP566N034 *	Hypothetical protein LOC81615	−0.04 (0.3)	10.8	2.9*E* − 06
cg17589341	18	*SLC14A1 *	Rsolute carrier family 14 (urea transporter), member 1	0.03 (0.3)	9.3	2.9*E* − 06
cg21126943	19	*CEACAM6 *	Carcinoembryonic antigen-related cell adhesion molecule 6 (nonspecific cross reacting antigen)	−0.8 (0.4)	6.9	5.7*E* − 06
cg07408456	19	*PGLYRP2 *	Peptidoglycan recognition protein L precursor	−0.2 (0.4)	8.3	5.7*E* − 06
cg25268718	14	*PSME1 *	Proteasome activator subunit 1 isoform 1	0.3 (0.2)	13.5	6.0*E* − 06
cg08700306	19	*LRP3 *	Low-density lipoprotein receptor-related protein 3	−0.04 (0.3)	9.7	6.1*E* − 06
cg02863947	3	*NR1I2 *	Pregnane X receptor isoform 2	−0.4 (0.5)	6.7	6.3*E* − 06

Model 1: eGFR_*ij*_ = *β*_0_ + *β*_1_ · CpG_*ij*_ + *W*_*j*_ + *ε*_*ij*_.

**Table 3:** Univariable and multivariable linear regression for eGFR.

Risk factors (*N* = 972)	Univariable model (Model 4)^a^		Multivariable model (Model 5)^b^	
	*β*_1_ (*P* value)	*R* ^2^	*β*_*k*_ (*P* value)	*R* ^2^
Homocysteine (*μ*mol/L)	−2.0 (4.9*E* − 48)	0.1965	−1.7 (7.6*E* − 36)	0.2827
Age (years)	−1.0 (8.9*E* − 35)	0.1445	−0.8 (1.1*E* − 21)	
Fibrinogen (mg/dL)	−0.03 (0.0009)	0.0114	−0.02 (0.0160)	
Serum cholesterol (mg/dL)	−0.06 (0.0002)	0.0140	−0.03 (0.0263)	
Sex^c^	2.8 (0.0560)	0.0038	—	—
Height (cm)	−0.1 (0.1137)	0.0026	—	—
Weight (kg)	−0.03 (0.4851)	0.0005	—	—
Waist circumference (cm)	−0.06 (0.1834)	0.0018	—	—
Hip circumference (cm)	−0.01 (0.9113)	0.0000	—	—
Body mass index (kg/m^2^)	0.02 (0.8402)	0.0000	—	—
C-reactive protein (mg/L)	0.5 (0.3814)	0.0008	—	—
Serum glucose (mg/dL)	0.02 (0.1869)	0.0018	—	—
Serum triglycerides (mg/dL)	−0.03 (0.0150)	0.0061	—	—
Systolic blood pressure (mm Hg)	−0.08 (0.0072)	0.0074	—	—
Diastolic blood pressure (mm Hg)	0.09 (0.1217)	0.0025	—	—
Combined high density lipoprotein (mg/dL)	0.02 (0.5416)	0.0004	—	—
Hypertension^d^	−6.4 (0.0002)	0.0141	—	—
Type 2 diabetes^e^	−1.2 (0.3992)	0.0007	—	—

^a^Model 2: eGFR_*i*_ = *β*_0_ + *β*_1_ · Risk  Factor_*i*_ + *ε*_*i*_.

^b^Model 5: eGFR_i_ = β_0_ + ∑_*m*=1_^*p*^ *β*_*m*_ · Risk  Factor_*mi*_ + ε_i_, where *p* is the number of risk factors.

^c^Female = 1, male = 0.

^d^Hypertension = 1, no hypertension = 0.

^e^Type 2 diabetes = 1, no type 2 diabetes = 0.
