# Supplementary material for: Epigenetic Markers of Renal Function in African Americans
Source: Nurs Res Pract. 2013 Dec 12;2013:687519. doi: 10.1155/2013/687519 (PMC3874945; doi:10.1155/2013/687519)
Supplement: Supplementary file 1 — The supplementary material includes five tables and two figures that further characterize the relationship between DNA methylation markers, traditional risk factors for kidney function, and estimated glomerular filtration rate (eGFR). Specifically, the tables show the correlation between the 30 methylation sites most strongly associated with eGFR (Supplementary Table 1), the relationship between these 30 methylation sites and the four risk factors that are most strongly associated with eGFR (Supplementary Table 2), the variation in eGFR that is explained by each of the 30 methylation sites after adjusting for each risk factor individually (Supplementary Table 3), the variation in eGFR that is explained by each of the 30 methylation sites after adjusting for all significant risk factors (Supplementary Table 4), and the variation in eGFR that is explained by the final model that includes the significant risk factors and methylation sites (Supplementary Table 5). Supplementary Figure 1 shows the distribution of the M-value for the 26,428 DNA methylation sites that were tested for association with eGFR. Supplementary Figure 2 shows the quantile-quantile plot of the association between each of the 26,428 DNA methylation sites and eGFR, without adjustment for any risk factors. [file 687519.f1.docx]

**SUPPLEMENTARY MATERIAL**

**Supplementary Figure 1. Distribution of mean M-Values across 26,428 methylation markers for 972 African Americans from the GENOA study.**

M-Value < -2 indicates unmethylated CpG sites

M-Value > +2 indicates methylated CpG sites

**Supplementary Figure 2. QQ-plot of 26,428 methylation marker p-values for eGFR using linear mixed modeling (Bonferonni alpha level = 0.05/26,248 = 1.89E-06).**

**Supplementary Table 1. Pearson’s correlation coefficients among top 30 CpG sites most strongly associated with eGFR.**

| CpG Site | 1 | 2 | 3 | 4 | 5 | 6 | 7 | 8 | 9 | 10 | 11 | 12 | 13 | 14 | 15 | 16 | 17 | 18 | 19 | 20 | 21 | 22 | 23 | 24 | 25 | 26 | 27 | 28 | 29 |
| --- | --- | --- | --- | --- | --- | --- | --- | --- | --- | --- | --- | --- | --- | --- | --- | --- | --- | --- | --- | --- | --- | --- | --- | --- | --- | --- | --- | --- | --- |
| 2 |  |  |  |  |  |  |  |  |  |  |  |  |  |  |  |  |  |  |  |  |  |  |  |  |  |  |  |  |  |
| 3 |  |  |  |  |  |  |  |  |  |  |  |  |  |  |  |  |  |  |  |  |  |  |  |  |  |  |  |  |  |
| 4 |  |  |  |  |  |  |  |  |  |  |  |  |  |  |  |  |  |  |  |  |  |  |  |  |  |  |  |  |  |
| 5 | - |  |  |  |  |  |  |  |  |  |  |  |  |  |  |  |  |  |  |  |  |  |  |  |  |  |  |  |  |
| 6 |  | - | - | - | - |  |  |  |  |  |  |  |  |  |  |  |  |  |  |  |  |  |  |  |  |  |  |  |  |
| 7 | - |  |  |  |  | - |  |  |  |  |  |  |  |  |  |  |  |  |  |  |  |  |  |  |  |  |  |  |  |
| 8 | - |  |  |  |  | - |  |  |  |  |  |  |  |  |  |  |  |  |  |  |  |  |  |  |  |  |  |  |  |
| 9 | - |  |  |  |  | - |  |  |  |  |  |  |  |  |  |  |  |  |  |  |  |  |  |  |  |  |  |  |  |
| 10 | - |  |  |  |  | - |  |  |  |  |  |  |  |  |  |  |  |  |  |  |  |  |  |  |  |  |  |  |  |
| 11 | - |  |  |  |  | - |  |  |  |  |  |  |  |  |  |  |  |  |  |  |  |  |  |  |  |  |  |  |  |
| 12 | - |  |  |  |  | - |  |  |  |  |  |  |  |  |  |  |  |  |  |  |  |  |  |  |  |  |  |  |  |
| 13 | - |  |  |  |  | - |  |  |  |  |  |  |  |  |  |  |  |  |  |  |  |  |  |  |  |  |  |  |  |
| 14 | - |  |  |  |  | - |  |  |  |  |  |  |  |  |  |  |  |  |  |  |  |  |  |  |  |  |  |  |  |
| 15 |  | - | - | - | - |  | - | - | - | - | - | - | - | - |  |  |  |  |  |  |  |  |  |  |  |  |  |  |  |
| 16 | - |  |  |  |  | - |  |  |  |  |  |  |  |  | - |  |  |  |  |  |  |  |  |  |  |  |  |  |  |
| 17 | - |  |  |  |  | - |  |  |  |  |  |  |  |  | - |  |  |  |  |  |  |  |  |  |  |  |  |  |  |
| 18 | - |  |  |  |  | - |  |  |  |  |  |  |  |  | - |  |  |  |  |  |  |  |  |  |  |  |  |  |  |
| 19 | - |  |  |  |  | - |  |  |  |  |  |  |  |  | - |  |  |  |  |  |  |  |  |  |  |  |  |  |  |
| 20 |  | - | - | - | - |  | - | - | - | - | - | - | - | - |  | - | - | - | - |  |  |  |  |  |  |  |  |  |  |
| 21 | - |  |  |  |  | - |  |  |  |  |  |  |  |  | - |  |  |  |  | - |  |  |  |  |  |  |  |  |  |
| 22 |  | - | - | - |  | -† | - |  |  | - | - | - | - | - |  | - | - | - | - |  |  |  |  |  |  |  |  |  |  |
| 23 |  | - | - |  | - |  | - | - | - | - | - | - | - | - |  | - |  | - | - |  | - |  |  |  |  |  |  |  |  |
| 24 | - |  |  |  |  | - |  |  |  |  |  |  |  |  | - |  |  |  |  | - |  | - | - |  |  |  |  |  |  |
| 25 | - |  |  |  |  | - |  |  |  |  |  |  |  |  | - |  |  |  |  | - |  | - | - |  |  |  |  |  |  |
| 26 | - |  |  |  |  | - |  |  |  |  |  |  |  |  | - |  |  |  |  | - |  | - | - |  |  |  |  |  |  |
| 27 | - |  |  |  |  | - |  |  |  |  |  |  |  |  | - |  |  |  |  | - |  | - | - |  |  |  |  |  |  |
| 28 | - |  |  |  |  | - |  |  |  |  |  |  |  |  | - |  |  |  |  | - |  | - | - |  |  |  |  |  |  |
| 29 | - |  |  |  |  | - |  |  |  |  |  |  |  |  | - |  |  |  |  | - |  | - | - |  |  |  |  |  |  |
| 30 | - |  |  |  |  | - |  |  |  |  |  |  |  |  | - |  |  |  |  | - |  | - | - |  |  |  |  |  |  |

† indicates the correlation for the site is not significant at 0.05.

- indicates that the correlation ‘r’ is negative.

Yellow indicates a correlation ‘r’ value of |0.00-0.20|;

Orange indicates a correlation ‘r’ value of |0.21-0.40|;

Maroon indicates a correlation ‘r’ value of |0.41-0.60|;

Purple indicates a correlation ‘r’ value of |0.61-0.80|;

Blue indicates a correlation ‘r’ value of |0.81-1.00|.

**Supplementary Table 2.** **Univariable** **linear regression of significant risk factors on the top 30 CpG sites (Model 3)^a^.**

| CpG Site (N=972) | Fibrinogen, mg/dL | | Homocysteine, µmol/L | | Serum Cholesterol, mg/dL | | Age, years | |
| --- | --- | --- | --- | --- | --- | --- | --- | --- |
|  | **β_1_ (p-value)** | **R^2^** | **β_1_ (p-value)** | **R^2^** | **β_1_ (p-value)** | **R^2^** | **β_1_ (p-value)** | **R^2^** |
| cg00226923 | **19.8** (0.0010)** | 0.0111 | **2.1** (5.2E-10)** | 0.0390 | -1.6 (0.6140) | 0.0003 | **3.1****  **(1.4E-08)** | 0.0326 |
| cg17471102 | -2.7 (0.7580) | 0.0001 | **-1.7** (0.0003)** | 0.0136 | -3.4 (0.4355) | 0.0006 | **-9.8****  **(1.2E-37)** | 0.1560 |
| cg12261786 | **-21.9* (0.0156)** | 0.0060 | **-2.3** (4.2E-06)** | 0.0216 | -8.6 (0.0613) | 0.0036 | **-7.6****  **(1.5E-20)** | 0.0852 |
| cg10917602 | **-29.7** (8.5E-07)** | 0.0247 | **-1.3** (0.0001)** | 0.0153 | 0.09 (0.9766) | 0.000 | **-5.7****  **(3.2E-26)** | 0.1093 |
| cg04662594 | -7.8 (0.2644) | 0.0013 | **-1.6** (0.00004)** | 0.0171 | -6.829 (0.0564) | 0.0037 | **-7.6****  **(4.1E-34)** | 0.1418 |
| cg15121304 | **25.5** (0.0018)** | 0.0100 | **3.2** (1.1E-12)** | 0.0509 | 2.5 (0.5455) | 0.0004 | **6.6****  **(4.0E-19)** | 0.0791 |
| cg24857721 | -2.3 (0.7241) | 0.0001 | **-1.3** (0.0002)** | 0.0140 | -0.4 (0.9069) | 0.000 | **-4.4****  **(4.0E-14)** | 0.0573 |
| cg14688272 | 1.5 (0.8695) | 0.000 | **-2.7** (5.0E-08)** | 0.0302 | 6.9 (0.1271) | 0.0024 | **-5.4****  **(2.0E-11)** | 0.0454 |
| cg19761273 | -12.9 (0.1395) | 0.0022 | **-3.4** (1.9E-12)** | 0.0499 | 0.06 (0.9889) | 0.000 | **-11.2****  **(1.1E-49)** | 0.2027 |
| cg24092253 | -12.3 (0.1652) | 0.0020 | **-2.1** (0.00001)** | 0.0194 | 3.9 (0.3880) | 0.0008 | **-6.0****  **(4.4E-14)** | 0.0571 |
| cg10126923 | **-17.2** (0.0011)** | 0.0110 | **-1.6** (2.5E-08)** | 0.0315 | -2.6 (0.3352) | 0.0010 | **-4.4****  **(4.2E-20)** | 0.0833 |
| cg25538571 | -10.6 (0.2167) | 0.0016 | **-2.0** (0.00003)** | 0.0180 | -2.2 (0.6163) | 0.0003 | **-9.6 (1.5E-36)** | 0.1517 |
| cg11120551 | -11.8 (0.1017) | 0.0028 | **-1.9** (1.2E-06)** | 0.0241 | 1.3 (0.7179) | 0.0001 | **-7.1****  **(1.3E-28)** | 0.1193 |
| cg00563932 | -10.9 (0.1616) | 0.0020 | **-1.4** (0.0010)** | 0.0110 | 1.6 (0.6927) | 0.0002 | **-6.3****  **(2.9E-19)** | 0.0797 |
| cg16280667 | **30.2** (0.00008)** | 0.0160 | **2.4** (1.9E-08)** | 0.0320 | **7.7* (0.0484)** | 0.0040 | **5.2****  **(7.5E-14)** | 0.0561 |
| cg12125117 | **-21.7** (0.0002)** | 0.0146 | **-1.7** (9.6E-08)** | 0.0289 | -1.9 (0.5115) | 0.0004 | **-4.1****  **(6.0E-15)** | 0.0609 |
| cg01820374 | -14.1 (0.0880) | 0.0030 | **-2.1** (3.2E-06)** | 0.0221 | 0.2 (0.9589) | 0.000 | **-9.9****  **(2.0E-42)** | 0.1749 |
| cg09809672 | -7.4 (0.2247) | 0.0015 | **-0.8* (0.0117)** | 0.0065 | -1.3 (0.6726) | 0.0002 | **-5.7****  **(7.5E-26)** | 0.1078 |
| cg14859417 | **-30.3** (0.00007)** | 0.0163 | **-1.9** (0.00001)** | 0.0199 | -3.1 (0.4327) | 0.0006 | **-5.8****  **(7.8E-17)** | 0.0691 |
| cg18152830 | **31.9** (0.0005)** | 0.0126 | **2.7** (7.5E-08)** | 0.0294 | 1.3 (0.7719) | 0.0001 | **5.1****  **(8.1E-10)** | 0.0382 |
| cg08743392 | **-14.3* (0.0412)** | 0.0043 | **-2.1** (7.9E-08)** | 0.0293 | -5.2 (0.1490) | 0.0021 | **-6.8****  **(2.1E-27)** | 0.1143 |
| cg26842024 | **14.0* (0.0499)** | 0.0040 | **0.8* (0.0370)** | 0.0045 | -5.3 (0.1457) | 0.0022 | **3.0****  **(3.8E-06)** | 0.0218 |
| cg07426848 | **24.5** (0.0026)** | 0.0093 | **1.5** (0.0006)** | 0.0120 | -1.3 (0.7601) | 0.0001 | **1.6** (0.0353)** | 0.0046 |
| cg15297650 | **-18.8* (0.0405)** | 0.0043 | **-2.3** (4.2E-06)** | 0.0216 | 0.2 (0.9737) | 0.000 | **-9.1****  **(2.4E-28)** | 0.1182 |
| cg17589341 | -1.2 (0.8809) | 0.000 | **-1.5** (0.0005)** | 0.0123 | -0.2 (0.9661) | 0.000 | **-5.2****  **(5.4E-13)** | 0.0523 |
| cg21126943 | **-22.8** (0.0001)** | 0.0148 | **-1.8** (3.5E-08)** | 0.0309 | -3.5 (0.2520) | 0.0014 | **-4.9****  **(1.7E-19)** | 0.0807 |
| cg07408456 | **-23.4** (0.0014)** | 0.0105 | **-1.4** (0.0005)** | 0.0125 | -5.0 (0.1796) | 0.0019 | **-5.8****  **(4.7E-18)** | 0.0744 |
| cg25268718 | -13.7 (0.2506) | 0.0014 | **-2.5** (0.0001)** | 0.0149 | 7.2 (0.2373) | 0.0014 | **-11.3****  **(4.0E-26)** | 0.1089 |
| cg08700306 | **-36.1** (0.00003)** | 0.0182 | **-1.7** (0.0004)** | 0.0130 | -6.0 (0.1699) | 0.0019 | **-5.1****  **(9.7E-11)** | 0.0423 |
| cg02863947 | **-23.7** (0.00005)** | 0.0168 | **-1.5** (1.9E-06)** | 0.0231 | 0.9 (0.7611) | 0.0001 | **-3.9** (2.5E-13)** | 0.0537 |

^a^Model 3: *Risk Factor_i_ = β_0_ + β_1_·CpG_i_ +ε_i_*

^*^0.01<p<0.05, ^**^p<0.01

**Supplementary Table 3.** **Bivariable** **linear regression of eGFR on each significant risk factor and CpG site (Model 4)^a^, followed by the R^2^_CpG_ (R^2^ difference between Model 4 - Model 2)^b^ value for each CpG site after adjusting for each risk factor.**

| CpG Site (N=972) | Fibrinogen, mg/dL | | Homocysteine, µmol/L | | Serum Cholesterol, mg/dL | | Age, years | |
| --- | --- | --- | --- | --- | --- | --- | --- | --- |
|  | **β_2_ (p-value)** | **R^2^_CpG_** | **β_2_ (p-value)** | **R^2^_CpG_** | **β_2_ (p-value)** | **R^2^_CpG_** | **β_2_ (p-value)** | **R^2^_CpG_** |
| cg00226923 | **-8.5** (1.3E-08)** | 0.0324 | **-5.0** (0.0003)** | 0.0109 |  |  | **-5.9** (0.00003)** | 0.0153 |
| cg17471102 |  |  | **8.9** (4.0E-06)** | 0.0174 |  |  | 2.6 (0.2308) | 0.0013 |
| cg12261786 | **12.7** (1.4E-08)** | 0.0323 | **8.8** (0.00002)** | 0.0152 |  |  | **5.9** (0.0070)** | 0.0064 |
| cg10917602 | **7.7** (3.4E-07)** | 0.0262 | **5.8** (0.00002)** | 0.015 |  |  | 2.7 (0.0693) | 0.0029 |
| cg04662594 |  |  | **7.1** (6.1E-06)** | 0.0167 |  |  | 2.8 (0.1153) | 0.0022 |
| cg15121304 | **-10.6** (1.8E-07)** | 0.0274 | **-5.1** (0.0071)** | 0.0059 |  |  | **-4.7* (0.0161)** | 0.0051 |
| cg24857721 |  |  | **5.6** (0.0001)** | 0.0123 |  |  | **3.8* (0.0126)** | 0.0055 |
| cg14688272 |  |  | **7.3** (0.0003)** | 0.0109 |  |  | **7.2** (0.0006)** | 0.0103 |
| cg19761273 |  |  | **4.7* (0.0191)** | 0.0045 |  |  | -0.5 (0.8080) | 0 |
| cg24092253 |  |  | **6.5** (0.0012)** | 0.0086 |  |  | **4.6* (0.0285)** | 0.0042 |
| cg10126923 | **6.7** (2.4E-07)** | 0.0269 | **4.0** (0.0007)** | 0.0094 |  |  | **2.9* (0.0234)** | 0.0045 |
| cg25538571 |  |  | **7.6** (0.00008)** | 0.0127 |  |  | 1.8 (0.3932) | 0.0006 |
| cg11120551 |  |  | **5.4** (0.0010)** | 0.0089 |  |  | 2.0 (0.2702) | 0.0011 |
| cg00563932 |  |  | **6.8** (0.0001)** | 0.0123 |  |  | 3.3 (0.0800) | 0.0027 |
| cg16280667 | **-8.9** (2.9E-06)** | 0.0221 | **-5.0** (0.0039)** | 0.0068 | **-9.2** (1.2E-06)** | 0.0236 | **-4.5* (0.0139)** | 0.0053 |
| cg12125117 | **6.7** (3.8E-06)** | 0.0216 | **3.9** (0.0033)** | 0.0071 |  |  | **3.1* (0.0240)** | 0.0045 |
| cg01820374 |  |  | **6.8** (0.0003)** | 0.0107 |  |  | 0.7 (0.7256) | 0.0001 |
| cg09809672 |  |  | **6.3** (2.9E-06)** | 0.0179 |  |  | 2.4 (0.1129) | 0.0022 |
| cg14859417 | **8.5** (8.1E-06)** | 0.0201 | **5.6** (0.0012)** | 0.0085 |  |  | 3.5 (0.0579) | 0.0032 |
| cg18152830 | **-9.9** (0.00001)** | 0.0193 | **-5.4** (0.0090)** | 0.0056 |  |  | **-5.6** (0.0092)** | 0.006 |
| cg08743392 | **8.4** (1.5E-06)** | 0.0234 | **4.7** (0.0031)** | 0.0072 |  |  | 1.9 (0.2657) | 0.0011 |
| cg26842024 | **-8.1** (4.8E-06)** | 0.0211 | **-6.8** (0.00002)** | 0.0149 |  |  | **-5.5** (0.0011)** | 0.0094 |
| cg07426848 | **-8.2** (0.00005)** | 0.0167 | **-5.8** (0.0015)** | 0.0083 |  |  | **-7.2** (0.0001)** | 0.0129 |
| cg15297650 | **10.4** (4.7E-06)** | 0.0212 | **6.3** (0.0022)** | 0.0077 |  |  | 1.7 (0.4413) | 0.0005 |
| cg17589341 |  |  | **6.8** (0.0001)** | 0.0121 |  |  | **4.7* (0.0128)** | 0.0055 |
| cg21126943 | **6.4** (0.00002)** | 0.0181 | **3.3** (0.0153)** | 0.0048 |  |  | 2.0 (0.1756) | 0.0016 |
| cg07408456 | **7.6** (0.00003)** | 0.0174 | **5.4** (0.0012)** | 0.0087 |  |  | 2.4 (0.1786) | 0.0016 |
| cg25268718 |  |  | **9.5** (0.0004)** | 0.0103 |  |  | 3.1 (0.2885) | 0.001 |
| cg08700306 | **9.3** (0.00002)** | 0.0189 | **6.8** (0.0004)** | 0.0101 |  |  | **5.1* (0.0125)** | 0.0055 |
| cg02863947 | **6.0** (0.00004)** | 0.017 | **3.6** (0.0076)** | 0.0058 |  |  | 2.7 (0.0563) | 0.0032 |

*^a^Model 4: eGFR*_i_ *= β_0_ + β_1_·Risk Factor_i_ + β_2_·CpG_i_ +ε_i_*

^b^R^2^_CpG_ = R^2^_difference_ for (*Model 4 – Model 2):* (*eGFR_i_ = β_0_ + β_1_·Risk Factor_i_ + β_2_·CpG_i_ +ε_i_*) – (*eGFR_i_ = β_0_ + β_1_·Risk Factor_i_ +ε_i_*), based on partial F-test.

^*^0.01<p<0.05, ^**^p<0.01

**Supplementary Table 4.** **Multivariable** **linear regression of eGFR on all four significant risk factors and each individual CpG site (Model 6)^a^, followed by the R^2^_CpG_ (R^2^ difference) value for each CpG site after adjusting for the risk factors (Model 6 - Model 5)^b^.**

| CpG Site (N=972) | Outcome: Estimated Glomerular Filtration Rate, mL/min per 1.732 m^2^ | | |
| --- | --- | --- | --- |
|  | **β_5_ (p-value)** | **R^2^** | **R^2^_CpG_** |
| cg00226923 | **-3.1* (0.0189)** | 0.2867 | 0.0040 |
| cg17471102 | 2.2 (0.2611) | 0.2836 |  |
| cg12261786 | 3.4 (0.0930) | 0.2848 |  |
| cg10917602 | 1.6 (0.2536) | 0.2836 |  |
| cg04662594 | 1.8 (0.2732) | 0.2836 |  |
| cg15121304 | -0.5 (0.7961) | 0.2827 |  |
| cg24857721 | **2.8* (0.0488)** | 0.2855 | 0.0028 |
| cg14688272 | **4.5* (0.0211)** | 0.2866 | 0.0039 |
| cg19761273 | -3.9 (0.0584) | 0.2853 |  |
| cg24092253 | 2.6 (0.1813) | 0.2840 |  |
| cg10126923 | 0.9 (0.4406) | 0.2831 |  |
| cg25538571 | 0.8 (0.6813) | 0.2828 |  |
| cg11120551 | 0.4 (0.8031) | 0.2827 |  |
| cg00563932 | 2.5 (0.1449) | 0.2842 |  |
| cg16280667 | -1.1 (0.5104) | 0.2830 |  |
| cg12125117 | 0.9 (0.4799) | 0.2830 |  |
| cg01820374 | -0.5 (0.8129) | 0.2827 |  |
| cg09809672 | 2.4 (0.08030 | 0.2849 |  |
| cg14859417 | 1.3 (0.4547) | 0.2831 |  |
| cg18152830 | -1.9 (0.3472) | 0.2833 |  |
| cg08743392 | -0.3 (0.8650) | 0.2827 |  |
| cg26842024 | **-4.9** (0.0015)** | 0.2901 | 0.0074 |
| cg07426848 | **-4.8** (0.0057)** | 0.2883 | 0.0056 |
| cg15297650 | -0.1 (0.9488) | 0.2827 |  |
| cg17589341 | **3.5* (0.0409)** | 0.2858 | 0.0031 |
| cg21126943 | -0.4 (0.7800) | 0.2827 |  |
| cg07408456 | 1.0 (0.5411) | 0.2829 |  |
| cg25268718 | 2.0 (0.4682) | 0.2831 |  |
| cg08700306 | 2.9 (0.1287) | 0.2844 |  |
| cg02863947 | 0.8 (0.5649) | 0.2829 |  |

*^a^Model 6: eGFR_i_ = β_0_ +* $\sum_{m=1}^{p} \beta_{m}$*·Risk Factor_mi_ + β_p+1_·CpG_i_ +ε_i_*, where p is the number of risk factors.

^b^R^2^_CpG_ = R^2^_difference_ for (*Model 6 – Model 5):* (*eGFR_i_ = β_0_ +* $\sum_{m=1}^{p} \beta_{m}$*·Risk Factor_mi_ + β_p+1_·CpG_i_ +ε_i_*) – (*eGFR_i_ = β_0_ +*$\sum_{m=1}^{p} \beta_{m}$*·Risk Factor_mi_ +ε_i_*)

^*^0.01<p<0.05, ^**^p<0.01

**Supplementary Table 5. Final model relating all significant risk factors and three of the six significant CpG sites to eGFR following forward model selection (Model 7)^a^.**

| **Model (N=972):** | **R^2^** |
| --- | --- |
| **eGFR*_i_* = 155.5 -0.01· Fibrinogen*_i_* -1.6· Homocysteine*_i_* -0.04· Serum Cholesterol*_i_* -0.7· Age*_i_* -4.4· cg26842024*_i_* -4.4· cg07426848*_i_* + 3.5· cg17589341*_i_* +*ε_i_*** | 0.2977 |

*^a^Model 7: eGFR_i_ = β_0_ +* $\sum_{m=1}^{p} \beta_{m}$*·Risk Factor_mi_ +* $\sum_{q=p+1}^{p+r} \beta_{q}$*·CpG_qi_ +ε*_i_, where p is the number of risk factors and r is the number of CpG sites.
